# A physiological signal database of children with different special needs for stress recognition

**DOI:** 10.1038/s41597-023-02272-2

**Published:** 2023-06-14

**Authors:** Buket Coşkun, Sevket Ay, Duygun Erol Barkana, Hilal Bostanci, İsmail Uzun, Ayse Betul Oktay, Basak Tuncel, Devrim Tarakci

**Affiliations:** 1 Yeditepe University, Faculty of Engineering, Department of Electrical and Electronics Engineering, Istanbul, 34755 Turkey; 2 INOSENS Bilisim, Gebze, Kocaeli, 41400 Turkey; 3 Istanbul Medipol University, Faculty of Health Sciences, Department of Ergotherapy, Istanbul, 34810 Turkey; 4 Yildiz Technical University, Faculty of Engineering, Department of Computer Engineering, Istanbul, 34349 Turkey

**Keywords:** Scientific data, Electrical and electronic engineering

## Abstract

This study presents a new dataset AKTIVES for evaluating the methods for stress detection and game reaction using physiological signals. We collected data from 25 children with obstetric brachial plexus injury, dyslexia, and intellectual disabilities, and typically developed children during game therapy. A wristband was used to record physiological data (blood volume pulse (BVP), electrodermal activity (EDA), and skin temperature (ST)). Furthermore, the facial expressions of children were recorded. Three experts watched the children’s videos, and physiological data is labeled “Stress/No Stress” and “Reaction/No Reaction”, according to the videos. The technical validation supported high-quality signals and showed consistency between the experts.

## Background & Summary

Serious games differ from ordinary games, which can be adjusted based on user/patient situations. For example, minimum/maximum angles for joints can be defined while playing a game which is very important for disabled people who need physical exercises. In the meantime, it is possible to get measurement data in serious games like balance, range of motion (ROM) etc. Serious games focus on patients’ required motions with cognitive actions, while ordinal games focus on entertainment.

Serious games are promising tools to improve upper extremity functions in children with neurological disorders. These games are developed as a rehabilitation program option to provide an exercise environment where children with different special needs can do many repetitions and receive immediate feedback during rehabilitation. Serious games have previously been shown to improve cognitive functions and motor skills such as attention, hand-eye coordination, and visual perception and facilitate learning^1–5^. In addition, games positively improve motivation, attention, processing speed, concentration, and visual discrimination in neurodevelopmental disorders such as dyslexia and mental retardation^5,6^.

Game therapies are more effective than traditional physiotherapy programs in improving upper extremity functions in children with Obstetric Brachial Plexus^7^. Furthermore, serious games-based rehabilitation has increased the motivation of children with dyslexia^8^. Children’s motivation during game therapy is essential, which may lead children to withdraw attention and even drop out of therapy. If a child gets stressed during game therapy, he/she can lose his/her motivation to continue the therapy. Thus, game therapies can be more prosperous if they recognize the children’s emotions/stress and modify the games considering their emotions/stress to increase their motivation and involvement.

Over the last decade, many multimodal emotion recognition databases containing data of adults have been published^9–22^. Even though many adult databases have been published, few emotion recognition databases including children have been developed. The emotion/stress detection of children is recently attracting considerable interest^23,24^, especially in children suffering from neurodevelopmental disorders^9,25–28^. Facial expression databases of typically developed children have been developed,i.e. CAFE^29^, NIMH-ChEFS^30^, Radboud Faces Database^31^, Dartmouth Database of Children’s Faces^32^, CEPS^33^, ChildEFES^34^. Furthermore, multimodal emotion recognition databases have been published for typically developing children containing audio, video, and physiological signals called EmoReact^23^ and MMDB^35^. Note that to our knowledge, no existing datasets are available that contain physiological signals and facial expressions of the children with different special needs (obstetric brachial plexus injury, dyslexia, and mental retardation) to be used for stress/emotion recognition of them.

AKTIVES dataset consists of physiological signals and camera recordings of 25 children with different special needs (obstetric brachial plexus injury, dyslexia, and mental retardation) and typically developed children who played serious games. The physiological signals (blood volume pulse (BVP), electrodermal activity (EDA), and skin temperature (ST)) were collected using the wearable Empatica E4 wristwatch^36^. Three experts annotated whether the children were stressed or not stressed and whether the children were given a reaction or no reaction through the game therapy. The dataset contains the video files recorded by the camera in Mp4 format. The videos include the child’s face and upper body during the game therapy.

AKTIVES dataset may contribute to the emotion recognition and stress detection models using physiological signals and facial expressions for children with different special needs. In addition, the AKTIVES dataset may serve to tackle the research questions related to (1) the multimodal approach to stress detection using physiological signals, (2) emotion recognition using facial expressions, and (3) the reaction of children to the developed serious games.

## Methods

### Ethics statement

Ethical approval has been obtained from the Research Ethics Committee of Istanbul Medipol University (NoE-10840098-772.02-6580). Furthermore, the parents of the children have been informed about the experimental procedure for the test and data acquisition setup and signed written consent. An informed consent form is used to inform parents about how the videos of children will be shared.

### Participants

A total of 25 children with obstetric brachial plexus injury, dyslexia, intellectual disabilities, and typically developed children participated in the study. In addition, 3 children with obstetric brachial plexus injury, 7 with intellectual disabilities, 4 with dyslexia, and 11 typically developed children participated in the study. The mean age of the children (10 males, 15 females) was 10.2 ± 1.27 years. Inclusion criteria consisted of a diagnosis of obstetric brachial plexus injury, intellectual disability, or dyslexia, aged between 5 to 14 years. Children with other chronic diseases were excluded from the study. Demographic and diagnostic characteristics of the participants were summarized in Table 1. The disability rates provided are 20–39 (individuals with special requirements), 40–49 (individuals with slight special requirements), 60–69 (individuals with advanced special needs), and 70–79 (individuals with very advanced special needs).

**Table 1:** Demographic and diagnostic characteristics of participants.

Participant	Gender	Age	Diagnosis	AoD	Disability Rate
C1	Female	13	OBPI	0	40–49
C2	Female	12	OBPI	0	60–69
C3	Male	10	Dyslexia	7	40–49
C4	Female	11	Dyslexia	8	20–39
C5	Female	11	Dyslexia	7	20–39
C6	Female	6	Dyslexia	4	20–39
C7	Male	12	Dyslexia	9	20–39
C8	Male	11	ID	4	20–39
C9	Female	12	ID	3	20–39
C10	Male	14	ID	4	60–69
C11	Male	5	ID	4	40–49
C12	Female	13	ID	3	40–49
C13	Male	11	ID	3	20–39
C14	Male	13	ID	3	60–69
C15	Male	12	ID	3	70–79
C16	Male	11	TD	—	—
C17	Female	5	TD	—	—
C18	Female	6	TD	—	—
C19	Female	8	TD	—	—
C20	Female	11	TD	—	—
C21	Female	12	TD	—	—
C22	Male	5	TD	—	—
C23	Male	5	TD	—	—
C24	Male	6	TD	—	—
C25	Female	10	TD	—	—

AoD: Age of Diagnosis, OBPI: Obstetric Brachial Plexus Injury, ID: Intellectual Disabilities, TD: Typically Developing. Disability rates for children are given by taking the rates of the Special Needs Report for Children reported by doctors in Turkey.

### Games

**Fig. 1:**
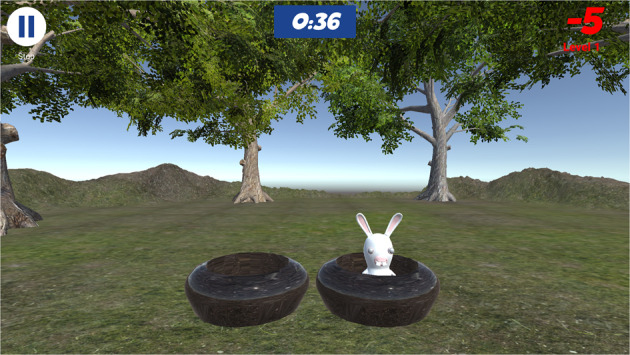
Becure CatchAPet.

**Fig. 2:**
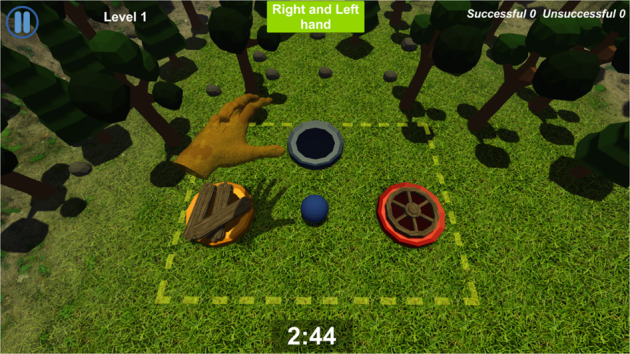
Becure LeapBall.

Becure CatchAPet and Becure LeapBall serious games were used in this study. First, the child tries to touch the rabbit on the screen in the playground with his hand in the virtual environment in the Becure CatchAPet game (Fig. 1). The child earns points for each contact with the rabbit and loses points for each missed rabbit. The child uses his/her wrist flexion/extension movement during the game. The wrist angles are determined by the physical therapists so that each child can virtually touch a rabbit with the correct range of motion. In the Becure LeapBall game, the child tries to drop the ball on the screen into buckets of the same color (Fig. 2). The child tries to hold and release the ball with grasping movements. The child directs the ball in the virtual environment, and if the child throws it into the correct bucket, he/she earns scores. The fail score increases when the child throws in the wrong basket. Various measures are recorded at the end of each game on the Becure E-Therapy web portal.

### Annotation

The children’s videos were recorded while playing Becure CatchAPet and Becure LeapBall. They were watched by three experts and were annotated as “Stress/No Stress” and “Reaction/No Reaction” every ten seconds. The children’s stress situations were assessed using children’s body language and annotated as “Stress/No Stress” and “Reaction/No Reaction”. The main manifestations of anxiety and stress on the human face involve the eyes (gaze distribution, blinking rate, pupil size variation), the mouth (mouth activity, lip deformations), and the cheeks, as well as the behavior of the head as a whole (head movements, head velocity). Additional facial signs of anxiety and stress in children may include a strained face, facial pallor, and eyelid twitching. All three experts are occupational therapists. Meanwhile, experts had at least 2 years of experience as an occupational therapist. Three experts did not see each other’s annotation. The majority of annotation has been kept. For example, if two experts annotated as stressed and one as no, stress has been assigned.

### Apparatus

Various studies have been done on emotion recognition and stress detection using Empatica E4 wristbands^37–43^. Emotions via Empatica E4 wristband in children with hearing disabilities have previously been investigated^44^. Further, Empatica E4 has been utilized for emotion recognition in children with atypical or delayed development^45^. In addition, the sympathetic response of children with autism under stressors has been studied using an E4 wristband^46^. The Empatica E4 wristband was previously used for children with autism spectrum disorder (ASD)^46^. In this study, physiological signals blood volume pulse (BVP), electrodermal activity (EDA), and skin temperature (ST) of children with obstetric brachial plexus injury, dyslexia, intellectual disabilities, and typically developed were collected using an Empatica E4 wristband^36^. The sampling frequency of the BVP signal was 64 Hz, while the EDA and ST signal was 4 Hz^36^. The E4 wristband was firmly placed on the child’s wrist without cutting off the blood flow. The collected data were synchronized and downloaded via E4 Manager software. The children’s upper body and facial expressions were recorded using the camera. The web camera of the laptop computer (Lenovo G510 with i7 processor) where the Becure Software by INOSENS company runs was used to get videos. The videos were in Mp4 format and had 720p resolution with image frames of 1280×720 pixels. The recording began when the physical therapist started the games and automatically ended when the game ended.

### Procedure

The study took place in the Technology Laboratory at Medipol University. The children and their parents were informed about the experimental procedure upon arrival. Then parents signed the written consent. The occupational therapists asked children to play the games approximately 2–3 minutes before the experiment so that children could get familiar with them. The child was asked to avoid unnecessary movements and to cover their faces.Children were instructed to fixate their gaze on a black screen for 30 seconds, serving as the baseline condition. Following the baseline period, the children were engaged in a serious game, either “Becure CatchAPet” or “Becure LeapBall,” for 420 seconds. Subsequently, upon completion of the game, the black screen was again presented for an additional 30 seconds. The procedure was repeated independently for each game.

**Fig. 3:**
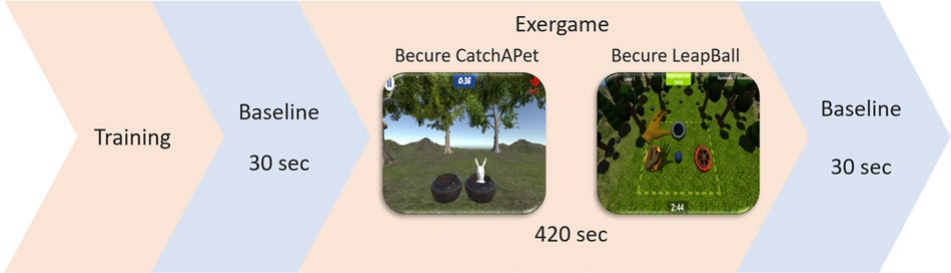
Experimental design.

**Fig. 4:**
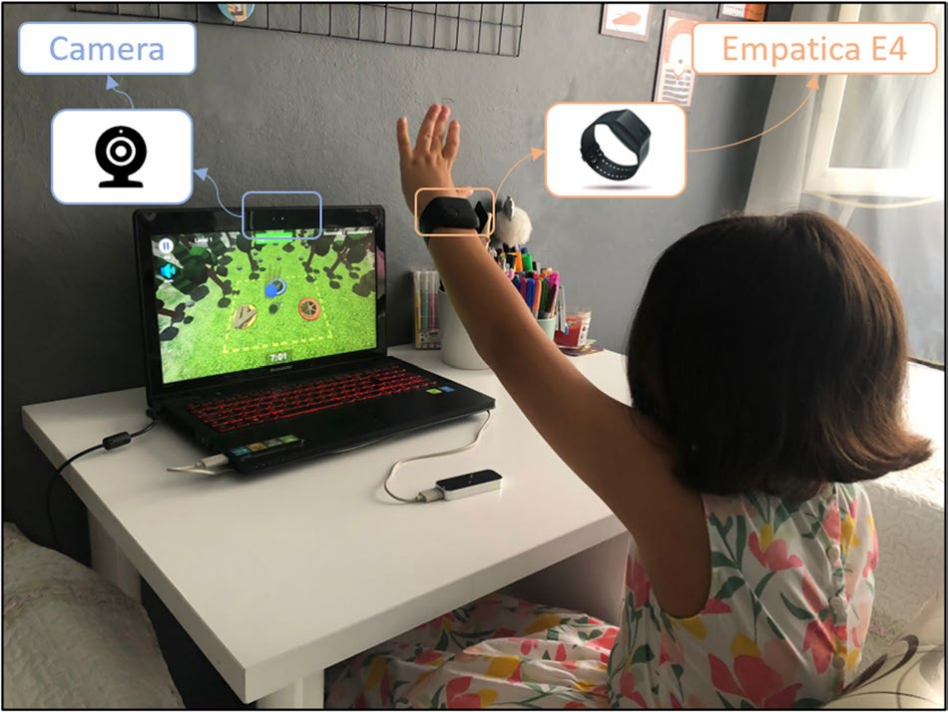
Experimental setup. The parents of the children signed written permission for the child’s picture to be included in this publication.

The experimental procedure is given in Fig. 3. The child’s physiological signal and facial expression were recorded during the session. In this order, each child was asked to do both Becure CatchAPet and Becure LeapBall games. The experimental setup is presented in Fig. 4.

### Data processing

The entire experimental setup was collected in a single device to ensure ideal data synchronization during the experiment. The E4 wristband was connected to the E4 Manager, and the device’s clock was synchronized before the experiment. E4 Manager was installed on the device on which the experimental setup was run. The clock update of the device presenting the experiment was checked. E4 was saved as a Unix timestamp when data recording started. The device’s clock was kept up to date, so this timestamp was synchronized with the whole mechanism. During the experiment, synchronous timestamps with stimuli were collected from all devices. The recording start or end (and video duration) timestamp of this video (creation time may not give correct results) were recorded in the metadata of the recorded video for labels to be synchronized with the physiological data. The face video recording was taken from the device on which the experimental setup was operated to ensure synchronization. While the data was kept separately for each client in Plain Text (.txt) format before preprocessing, it was stored in Comma-Separated Values (.csv) format (tabular data) after preprocessing.

A sixth-order Chebyshev II filter with the 18 dB stopband attenuation (Rs) and 0.1 Hz normalized stopband edge frequency (Wn) was used to preprocess the blood volume pulse (BVP) data^47^. The electrodermal activity (EDA) signal was filtered using a fifth-order Savitzky-Golay filter with a frame length parameter equal to 11^39^. Note that the two individuals that express the same emotions might have physiological signals with different levels^40^. Thus, the BVP and EDA signals were normalized between 0 and 100.

The main purpose of this study is to provide a dataset consisting of physiological signals labeled in terms of “Stress/ No Stress” by the experts. However, the face landmarks and facial emotions of children detected with well-known methods were also given in the AKTIVES dataset for researchers to investigate the correlation between gaming motivation and facial emotions for future research. The videos had a 9.7 frame rate, and the videos’ duration depended on the children’s gaming performance. The total number of frames in a video file was nearly 4300. The emotions of the children were also recognized using their faces. Initially, the faces of the children were detected using Multi-Task Cascaded Convolutional Neural Networks (MTCNN)^48^. Then, the emotions were recognized using an open dataset called The Facial Emotion Recognition (FER 2013)^49^. FER 2013 dataset included 48 × 48 pixel grayscale images of faces. Each face was labeled with seven categories (0 = Angry, 1 = Disgust, 2 = Fear, 3 = Happy, 4 = Sad, 5 = Surprise, 6 = Neutral). Note that the FER dataset was previously trained on children and adults, and 75.42% accuracy was obtained^50^.

## Data Records

**Fig. 5:**
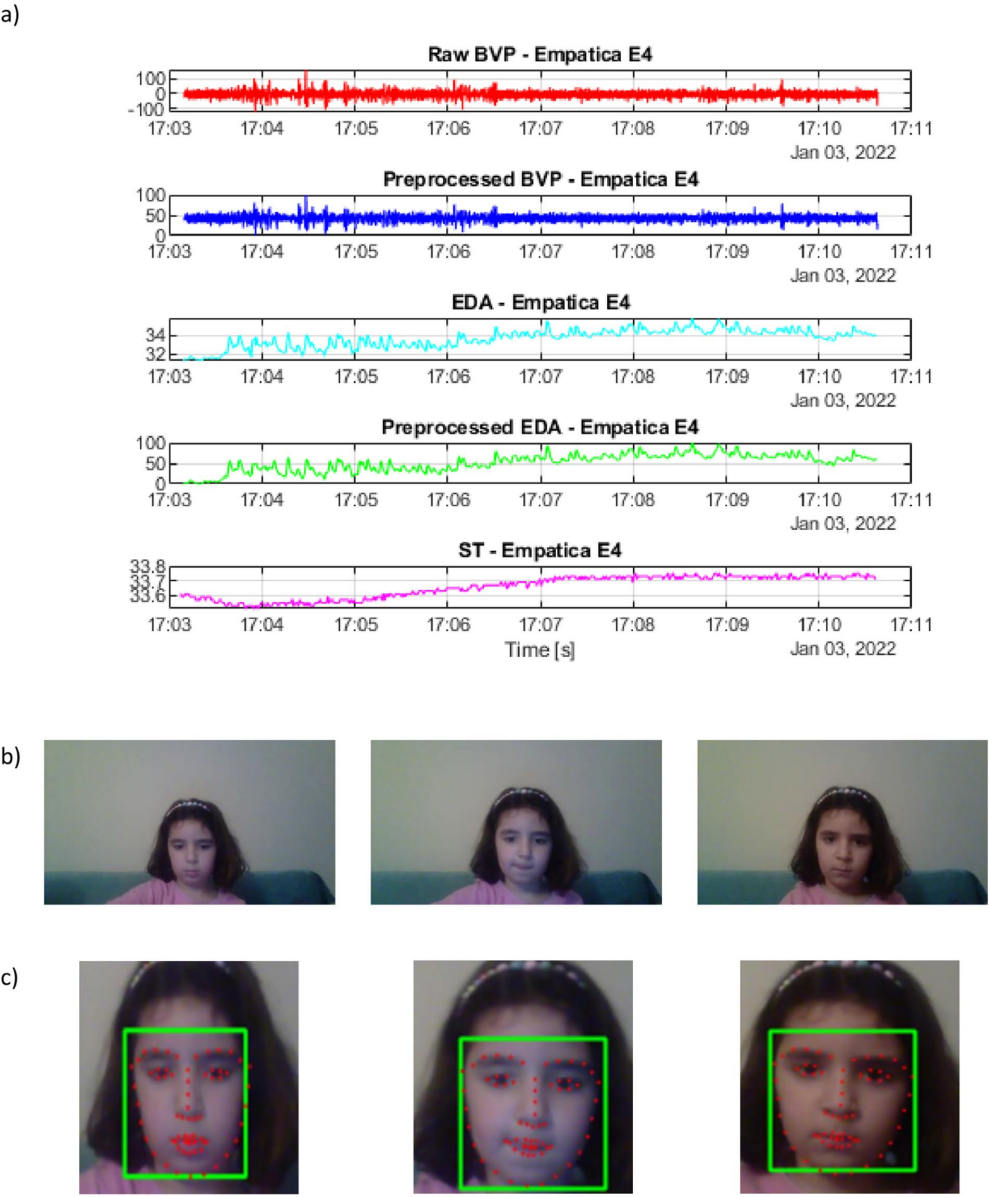
Examples of data available in the AKTIVES Dataset; (**a**) Physiological signals recorded with Empatica E4: BVP, EDA, ST (**b**) 3 sample video frames of a child (**c**) The facial emotions are detected as neural for the first two columns and sad for the third column with FER library. The faces are cropped for better visualization, and the facial landmarks are generated with the Dlib PyPI library. The children’s parents gave written consent to include her image in this publication.

The data recordings (physiological signals, camera recordings, expert annotations) are available at Synapse | Sage Bionetworks^51^. Figure 5 illustrates the physiological signal and video recording examples. The data is provided in four categories depending on the child’s condition (Obstetric Brachial Plexus Injury, Dyslexia, Intellectual Disabilities, Typically Developed). The child condition folders include the directories given names with each child’s ID (C1, C2, etc.). These child ID folders comprised two subfolders with serious game names Becure CatchAPet, and Becure LeapBall. The annotations of the experts (ExpertLabels.csv) in Comma-Separated Values (.csv) format, physiological (BVP, EDA, ST) and acceleration (ACC) data in a Comma-Separated Values (.csv) files, and camera data available in MPEG-4 (.mp4) format are all included in each serious games subfolders. In serious game subfolders, the automatically detected facial expressions from videos are also given in Comma-Separated Values (.csv) format (facial.csv). The full size of the data included in a repository is 4.94 GB. In total, there are 7 files for a child. These files and their descriptions are summarized in Table 2.

**Table 2:** Data files and descriptions for each participant.

File Name	Description	Data Format	Column Names
ACC.csv	3-axis Accelerometer data	.csv	ACC: Data Name, time: Timestamp, x: x-axis Data, y: y-axis Data, z: z-axis Data
BVP.csv	Blood Volume Pulse data	.csv	BVP: Data Name, time: Timestamp, Values: BVP Data
EDA.csv	Electrodermal Activity data	.csv	EDA: Data Name, time: Timestamp, Values: EDA Data
ExpertLabels.csv	Stress and reaction annotations of the experts	.csv	index, Minute, Second, Expert 1 Stress/No Stress, Expert 1 Reaction/No Reaction, Expert 2 Stress/No Stress, Expert 2 Reaction/No Reaction, Expert 3 Stress/No Stress, Expert 3 Reaction/No Reaction,
Facial.csv	Child’s automatically detected facial expressions	.csv	index, box, angry, disgust, fear, happy, sad, surprise, neutral
ST.csv	Skin Temperature data	.csv	ST: Data Name, time: Timestamp, Values: ST Data
Video.mp4	Video recordings	.mp4	—

## Technical Validation

Three experts annotated “Stress/No Stress” and “Reaction/No Reaction” through the execution of the serious games. The percentage of matching annotations to all annotations was calculated to validate the experts’ annotations. The agreement percentage was calculated using Eq. 1. Two experts did the calculations. (Experts 1–2, Experts 1–3, Experts 2–3) and all three experts (Experts 1-2-3).

1 $$Agreement=\frac{\#same\;annotation}{\#timestamps}$$

The maximum percentage for the “Stress/No Stress” annotations between two experts was 81.45%, while the minimum was 77.24%. The percentage agreement between the three annotations was 68.18% for the “Stress/No Stress”. The minimum result for the “Reaction/No Reaction” annotations was 69.50% between two experts, and the maximum was 83.24%. The agreement results for all experts were calculated as 63.39%. The results are presented in Table 3.

**Table 3:** The expert agreement results with respect to “Stress/ No Stress” and “Reaction/ No Reaction”.

	Experts 1-2	Experts 1–3	Experts 2–3	Experts 1-2-3
Stress/No Stress	81.45%	77.24%	77.68%	68.18%
Reaction/No Reaction	83.24%	69.50%	73.56%	63.39%

The signal-to-noise ratio (SNR) of the physiological signals acquired via the Empatica E4 device was calculated to validate the signal quality. The SNR metric was obtained with the help of the autocorrelation function utilizing a second-order polynomial to fit the autocorrelation function curve^52^. Whole physiological data were examined separately in the case of SNR. Raw physiological signals were utilized for the SNR estimation. Table 4 gave detailed statistical results of the SNR values. The Blood Volume Pulse (BVP) signal mean SNR was 33.44 dB with a 2.48 dB standard deviation, while the median (Q50) was 32.94 dB. The SNRs of the BVP signal change were between 29.56 dB to 39.31 dB. The mean SNR of the Electrodermal Activity (EDA) was 32.56 dB with a 2.38 dB standard deviation. The EDA signal’s median (Q50) SNR was 33.01 dB. The maximum SNR of the EDA signal was 37.62 dB, while the minimum was 25.07 dB. The estimated mean SNR for the Skin Temperature (ST) was 33.22 dB with a 2.05 dB standard deviation. The ST signal’s median (Q50) SNR value was 34.28 dB. There was no value below 26.46 dB or above 34.37 dB for the SNRs of the ST signal. Furthermore, subgroup statistics can be found in Table 4. In agreement with SNR findings, the physiological data acquired from Empatica E4 were good-quality signals.

**Table 4:** Signal to Noise Ratio (SNR) statistics of the physiological signal acquired via Empatica E4 wristband.

Condition	Serious Game	Signal	Mean	Std	Min	Q15	Q25	Q50	Q75	Q95	Max
OBPI	Becure CatcAPet	BVP	31.73	0.59	31.13	31.31	31.43	31.73	32.02	32.26	32.32
		EDA	32.33	2.44	29.89	30.62	31.11	32.33	33.55	34.53	34.77
		ST	33.02	1.30	31.72	32.11	32.37	33.02	33.67	34.19	34.32
	Becure LeapBall	BVP	32.08	0.49	31.60	31.74	31.84	32.08	32.33	32.52	32.57
		EDA	31.23	0.79	30.44	30.67	30.83	31.23	31.62	31.94	32.01
		ST	33.06	1.08	31.98	32.31	32.52	33.06	33.60	34.03	34.14
Dislexia	Becure CatcAPet	BVP	33.03	2.80	29.81	30.78	31.43	32.41	34.00	36.79	37.48
		EDA	30.46	2.72	26.17	27.91	29.08	31.29	32.67	32.99	33.07
		ST	31.12	2.62	26.79	28.87	30.26	32.01	32.87	33.50	33.66
	Becure LeapBall	BVP	33.57	2.52	30.52	31.03	31.37	33.74	35.95	36.21	36.28
		EDA	32.15	1.11	30.47	31.12	31.55	32.36	32.96	33.34	33.44
		ST	33.38	0.58	32.67	32.87	33.00	33.30	33.68	34.14	34.25
ID	Becure CatcAPet	BVP	33.64	2.53	29.76	30.74	31.89	33.80	35.30	37.16	38.03
		EDA	32.46	2.86	25.07	32.56	32.78	33.29	34.04	34.35	34.45
		ST	33.17	2.55	26.46	33.56	33.72	34.29	34.32	34.35	34.37
	Becure LeapBall	BVP	35.48	2.91	30.90	31.48	33.71	36.28	37.61	38.71	39.31
		EDA	31.57	2.07	27.66	30.33	30.94	31.69	32.40	34.39	34.99
		ST	32.99	2.33	27.59	31.61	33.10	34.28	34.29	34.31	34.31
TD	Becure CatcAPet	BVP	32.81	2.10	29.56	31.18	31.46	32.99	33.76	36.14	37.06
		EDA	33.35	2.22	27.37	33.02	33.26	34.32	34.53	34.96	34.98
		ST	33.57	2.14	27.50	34.31	34.31	34.32	34.33	34.35	34.37
	Becure LeapBall	BVP	33.15	1.63	30.28	31.30	32.07	33.28	34.23	35.35	35.53
		EDA	33.95	1.39	32.24	32.77	33.10	33.89	34.14	36.15	37.62
		ST	33.97	0.45	33.14	33.38	33.55	34.28	34.29	34.31	34.32
Total		BVP	33.44	2.48	29.56	31.02	31.43	32.94	35.15	37.61	39.31
		EDA	32.56	2.38	25.07	30.46	31.84	33.01	34.14	34.97	37.62
		ST	33.22	2.05	26.46	32.04	33.19	34.28	34.31	34.33	34.37

OBPI: Obstetric Brachial Plexus Injury, ID: Intellectual Disabilities, TD: Typically Developing, BVP: Bloop Volume Pulse, EDA: Electrodermal Activity, ST: Skin Temperature. Q means quantiles. All the SNR values are given in decibels (dB).

## Usage Notes

### Stress recognition

The most common approach for stress recognition using physiological signals and facial expression includes (1) data collection, (2) signal preprocessing, synchronization, and integration, (3) feature extraction and selection, and (4) machine and deep learning training and validation. A comprehensive overview of all these stages can be found in review papers on stress/emotion detection using wearables^53–55^.

We recommend the following Python libraries for further processing the AKTIVES dataset, which we found useful for preprocessing and feature extraction from physiological data. *Numpy Library* (https://numpy.org) is utilized for feature extraction from BVP, EDA and ST signals. *SciPy Library* (https://scipy.org), which provides signal processing algorithms such as signal filtering, is used to filter the BVP and EDA signal. The *Pandas Library* (https://pandas.pydata.org) is used to resample the psychological signal every 10 seconds. *NeuroKit2 Library*^56^, which is a user-friendly package, (https://neuropsychology.github.io/NeuroKit) to extract the features of EDA signals. For the SNR calculation, the GitHub repository^52^ (https://github.com/psychosensing/popane-2021) was used.

The state-of-the-art face detection and emotion recognition include data collection, feature extraction, and machine and deep learning^57,58^. For the dataset, face detection and emotion recognition are implemented in Python programming language using the *Opencv Library* (https://opencv.org/). Emotion detection is performed using the *FER Library* (https://pypi.org/project/fer/). For facial landmarks, pre-trained facial landmark recognition in the *Dlib Library* (http://dlib.net/) is used.

### Accessing data

The AKTIVES dataset must be used for academic purposes only. Researchers who want to access the AKTIVES dataset must first sign the End User License Agreement (EULA). The EULA is available in the AKTIVES Dataset repository. To access AKTIVES Dataset, a signed EULA must be sent to aktives.project@gmail.com. Only e-mails sent from the academic e-mail address will be considered.

## Data Availability

The codes include preprocessing of physiological signals, annotation synchronization, facial expression detection, and technical validation available at the Repository for the AKTIVES Dataset 2022 GitHub repository https://github.com/hiddenslate/aktives-dataset-2022. The Python 3.9 version has been utilized for the development of algorithms. In the requirements.txt file, all necessary packages are mentioned. Uploaded codes can be helpful guidelines to preprocess and analyze the AKTIVES dataset.

## References

[1] Rutkowski, S. *et al*. Training using a commercial immersive virtual reality system on hand–eye coordination and reaction time in young musicians: A pilot study. *International Journal of Environmental Research and Public Health* **18** (2021). 10.3390/ijerph18031297 PMC7908336 33535539

[2] Avila-Pesantez D, Delgadillo R, Rivera LA (2019). Proposal of a conceptual model for serious games design: A case study in children with learning disabilities. IEEE Access.

[3] Raygoza-Romero, J., Gonzalez-Hernandez, A., Bermudez, K., Martinez-Garcia, A. I. & Caro, K. Move&learn: An adaptive exergame to support visual-motor skills of children with neurodevelopmental disorders. In *Proceedings of the Conference on Information Technology for Social Good*, 169–174 (Association for Computing Machinery, New York, NY, USA, 2021).

[4] Milajerdi, H. R., Ordooiazar, F. & Dewey, D. Is active video gaming associated with improvements in social behaviors in children with neurodevelopmental disorders: a systematic review. *Child Neuropsychology* 1–27 (2022). 10.1080/09297049.2022.2046721 35236234

[5] Tarakci E, Arman N, Tarakci D, Kasapcopur O (2020). Leap motion controller–based training for upper extremity rehabilitation in children and adolescents with physical disabilities: A randomized controlled trial. Journal of Hand Therapy.

[6] Snowling MJ, Hulme C, Nation K (2020). Defining and understanding dyslexia: past, present and future. Oxford Review of Education.

[7] El-Shamy S, Alsharif R (2017). Effect of virtual reality versus conventional physiotherapy on upper extremity function in children with obstetric brachial plexus injury. Journal of Musculoskeletal Neuronal Interactions.

[8] Adams R, Finn P, Moes E, Flannery K, Rizzo A (2009). Distractibility in attention deficit hyperactivity disorder (adhd): The virtual reality classroom. Child Neuropsychology.

[9] Saganowski, S. *et al*. Emognition dataset: emotion recognition with self-reports, facial expressions, and physiology using wearables. *Scientific Data* **9** (2022). 10.1038/s41597-022-01262-0 PMC8989970 35393434

[10] Jiang, X. *et al*. Dfew: A large-scale database for recognizing dynamic facial expressions in the wild. In *Proceedings of the 28th ACM International Conference on Multimedia*, 2881–2889 (2020).

[11] Cheng, S., Kotsia, I., Pantic, M. & Zafeiriou, S. 4dfab: A large scale 4d database for facial expression analysis and biometric applications. In *2018 IEEE/CVF Conference on Computer Vision and Pattern Recognition*, 5117–5126 (2018).

[12] Davison AK, Lansley C, Costen N, Tan K, Yap MH (2018). Samm: A spontaneous micro-facial movement dataset. IEEE Transactions on Affective Computing.

[13] Mollahosseini A, Hasani B, Mahoor MH (2019). AffectNet: A database for facial expression, valence, and arousal computing in the wild. IEEE Transactions on Affective Computing.

[14] Li, S., Deng, W. & Du, J. Reliable crowdsourcing and deep locality-preserving learning for expression recognition in the wild. In *2017 IEEE Conference on Computer Vision and Pattern Recognition (CVPR)*, 2584–2593 (2017).

[15] Zhang Z, Luo P, Loy CC, Tang X (2018). From facial expression recognition to interpersonal relation prediction. Int. J. Comput. Vision.

[16] Benitez-Quiroz, C. F., Srinivasan, R. & Martinez, A. M. Emotionet: An accurate, real-time algorithm for the automatic annotation of a million facial expressions in the wild. In *2016 IEEE Conference on Computer Vision and Pattern Recognition (CVPR)*, 5562–5570 (2016).

[17] Schmidt, P., Reiss, A., Duerichen, R., Marberger, C. & Van Laerhoven, K. Introducing wesad, a multimodal dataset for wearable stress and affect detection. In *Proceedings of the 20th ACM International Conference on Multimodal Interaction*, ICMI ‘18, 400–408 (Association for Computing Machinery, New York, NY, USA, 2018).

[18] Miranda-Correa JA, Abadi MK, Sebe N, Patras I (2021). Amigos: A dataset for affect, personality and mood research on individuals and groups. IEEE Transactions on Affective Computing.

[19] Koelstra S (2012). Deap: A database for emotion analysis;using physiological signals. IEEE Transactions on Affective Computing.

[20] Abadi MK (2015). Decaf: Meg-based multimodal database for decoding affective physiological responses. IEEE Transactions on Affective Computing.

[21] Ringeval, F., Sonderegger, A., Sauer, J. & Lalanne, D. Introducing the recola multimodal corpus of remote collaborative and affective interactions. In *2013 10th IEEE International Conference and Workshops on Automatic Face and Gesture Recognition (FG)*, 1–8 (2013).

[22] Soleymani M, Lichtenauer J, Pun T, Pantic M (2012). A multimodal database for affect recognition and implicit tagging. IEEE Transactions on Affective Computing.

[23] Nojavanasghari, B., Baltrušaitis, T., Hughes, C. E. & Morency, L.-P. Emoreact: A multimodal approach and dataset for recognizing emotional responses in children. In *Proceedings of the 18th ACM International Conference on Multimodal Interaction*, ICMI ‘16, 137–144 (Association for Computing Machinery, New York, NY, USA, 2016).

[24] Lopez-Rincon, A. Emotion recognition using facial expressions in children using the nao robot. In *2019 International Conference on Electronics, Communications and Computers (CONIELECOMP)*, 146–153 (2019).

[25] Sarabadani S, Schudlo LC, Samadani AA, Kushski A (2020). Physiological detection of affective states in children with autism spectrum disorder. IEEE Transactions on Affective Computing.

[26] Krupa, N., Anantharam, K., Sanker, M., Datta, S. & Kommu, J. V. S. Recognition of emotions in autistic children using physiological signals. *Health and Technology* **6** (2016).

[27] Tiinanen, S. *et al*. Hrv and eeg based indicators of stress in children with asperger syndrome in audio-visual stimulus test. In *2011 Annual International Conference of the IEEE Engineering in Medicine and Biology Society*, 2021–2024 (2011). 10.1109/IEMBS.2011.6090371 22254732

[28] Bairavi, K. & Sundhara, K. B. K. Eeg based emotion recognition system for special children. In *Proceedings of the 2018 International Conference on Communication Engineering and Technology*, ICCET ‘18, 1–4 (Association for Computing Machinery, New York, NY, USA, 2018).

[29] LoBue V, Thrasher C (2014). The child affective facial expression (cafe) set: Validity and reliability from untrained adults. Frontiers in psychology.

[30] Egger H (2011). The nimh child emotional faces picture set (nimh-chefs): a new set of children’s facial emotion stimuli. International journal of methods in psychiatric research.

[31] Langner O (2010). Presentation and validation of the radboud faces database. Cognition and Emotion.

[32] Dalrymple K, Gomez J, Duchaine B (2013). The dartmouth database of children’s faces: Acquisition and validation of a new face stimulus set. PloS one.

[33] Romani-Sponchiado A, Sanvicente-Vieira B, Mottin C, Hertzog D, Arteche A (2015). Child emotions picture set (ceps): Development of a database of children’s emotional expressions. Psychology & Neuroscience.

[34] Negrão, J. *et al*. The child emotion facial expression set: A database for emotion recognition in children. *Frontiers in Psychology* **12** (2021). 10.3389/fpsyg.2021.666245 PMC8116652 33995223

[35] Rehg, J. M. *et al*. Decoding children’s social behavior. In *2013 IEEE Conference on Computer Vision and Pattern Recognition*, 3414–3421 (2013).

[36] Empatica. *E4 wristband from empatica user’s manuel*. UM-16Rev.2.0 (2020).

[37] Chandra, V. *et al*. Comparative study of physiological signals from empatica e4 wristband for stress classification. In *Advances in Computing and Data Sciences*, 218–229 (Springer International Publishing, Cham, 2021).

[38] Sevil M (2020). Detection and characterization of physical activity and psychological stress from wristband data. Signals.

[39] Sevil M (2021). Discrimination of simultaneous psychological and physical stressors using wristband biosignals. Computer Methods and Programs in Biomedicine.

[40] Zhao, B., Wang, Z., Yu, Z. & Guo, B. Emotionsense: Emotion recognition based on wearable wristband. In *2018 IEEE SmartWorld, Ubiquitous Intelligence & Computing, Advanced & Trusted Computing, Scalable Computing & Communications, Cloud & Big Data Computing, Internet of People and Smart City Innovation (SmartWorld/SCALCOM/UIC/ATC/CBDCom/IOP/SCI)*, 346–355 (2018).

[41] Cosoli G, Poli A, Scalise L, Spinsante S (2021). Measurement of multimodal physiological signals for stimulation detection by wearable devices. Measurement.

[42] Gjoreski M, LuÅ¡trek M, Gams M, Gjoreski H (2017). Monitoring stress with a wrist device using context. Journal of Biomedical Informatics.

[43] Bulagang, A., Mountstephens, J. & Teo, J. Multiclass emotion prediction using heart rate and virtual reality stimuli. *Journal of Big Data* **8** (2021).

[44] Uluer, P., Kose, H., Gumuslu, E. & Barkana, D. E. Experience with an affective robot assistant for children with hearing disabilities. *International Journal of Social Robotics* (2021). 10.1007/s12369-021-00830-5 PMC8594648 34804256

[45] Redd, C. B. *et al*. Physiological signal monitoring for identification of emotional dysregulation in children. In *2020 42nd Annual International Conference of the IEEE Engineering in Medicine & Biology Society (EMBC)*, 4273–4277 (2020). 10.1109/EMBC44109.2020.9176506 33018940

[46] Gul Airij A, Sudirman R, Sheikh UU, Lee YK, Zakaria N (2020). Significance of electrodermal activity response in children with autism spectrum disorder. Indonesian Journal of Electrical Engineering and Computer Science.

[47] Elgendi, M. *PPG Signal Analysis: An Introduction Using MATLAB* (CRC Press, Taylor & Francis Group, 2021).

[48] Zhang K, Zhang Z, Li Z, Qiao Y (2016). Joint face detection and alignment using multitask cascaded convolutional networks. IEEE Signal Processing Letters.

[49] Goodfellow, I. J. *et al*. Challenges in representation learning: A report on three machine learning contests. *Neural Networks* **64**, 59–63 (2015). Special Issue on “Deep Learning of Representations”. 10.1016/j.neunet.2014.09.005 25613956

[50] Georgescu M-I, Ionescu RT, Popescu M (2019). Local learning with deep and handcrafted features for facial expression recognition. IEEE Access.

[51] Coskun B (2023). Synapse.

[52] Behnke, M., Buchwald, M., Bykowski, A., Kupiński, S. & Kaczmarek, L. Psychophysiology of positive and negative emotions, dataset of 1157 cases and 8 biosignals. *Scientific Data* **9** (2022). 10.1038/s41597-021-01117-0 PMC8776805 35058476

[53] Wang Y (2022). A systematic review on affective computing: emotion models, databases, and recent advances. Information Fusion.

[54] Giannakakis G (2022). Review on psychological stress detection using biosignals. IEEE Transactions on Affective Computing.

[55] Saganowski, S. Bringing emotion recognition out of the lab into real life: Recent advances in sensors and machine learning. *Electronics* **11** (2022).

[56] Makowski, D. *et al*. Neurokit2: A python toolbox for neurophysiological signal processing. *Behavior Research Methods* **53** (2021). 10.3758/s13428-020-01516-y 33528817

[57] Wu Y, Ji Q (2018). Facial landmark detection: A literature survey. International Journal of Computer Vision.

[58] Canal FZ (2022). A survey on facial emotion recognition techniques: A state-of-the-art literature review. Information Sciences.

